# BIOMARKERS OF AGING AND PRECISION HEALTH CARE FOR OLDER ADULTS

**DOI:** 10.1093/geroni/igae098.1908

**Published:** 2024-12-31

**Authors:** Anthony Molina

**Affiliations:** University of California San Diego, La Jolla, California, United States

## Abstract

Efforts to develop objective and quantifiable biomarkers of human aging are ongoing and have been accelerated by advances in technical and analytical approaches for biomedical research. Beyond predicting one’s chronological age, this research is being increasingly applied to measure biological age, which captures the heterogeneity of the aging process. Such markers may be able to predict one’s risk for developing a variety of age-related conditions and diseases and are recognized to have potential applications for advancing precision healthcare for older adults. This presentation will review the development of promising biomarkers of human aging and will provide examples of how markers are being tested and applied in a range of clinical studies. As approaches for promoting healthy longevity are being developed and evaluated, biomarkers of human aging may serve as important study outcomes. Modifiable biomarkers of human aging may reflect effects on healthspan and longevity, which would otherwise take many decades to monitor. Finally, this presentation will review national and international cooperative efforts to evaluate and compare biomarkers of human aging, Such efforts are also applying biomarker development approaches to complementary topics, such as the assessment of human resilience.

